# Anästhesie zur Sectio caesarea bei diastropher Dysplasie

**DOI:** 10.1007/s00101-024-01440-2

**Published:** 2024-08-05

**Authors:** Michaela Sieker, Thomas Weber, Heike Vogelsang, Peter Kern

**Affiliations:** 1 Klinik für Anästhesiologie und Intensivmedizin, Katholisches Klinikum Bochum, Universitätsklinikum der Ruhr-Universität Bochum, Gudrunstr. 56, 44891 Bochum, Deutschland; 2 Klinik für Gynäkologie und Geburtshilfe, St. Elisabeth-Hospital Bochum, Ruhr-Universität Bochum, Bochum, Deutschland

## Anamnese

Eine 34-jährige Patientin stellt sich in der 38 + 0 Schwangerschaftswoche [SSW] vor elektiver Sectio caesarea in der Prämedikationsambulanz vor. Die Patientin hatte den ausdrücklichen Wunsch einer Spinalanästhesie. Als Vorerkrankung berichtete sie von einer diastrophen Dysplasie. Im Alter von einem, 7 und 8 Jahren wurden bei Klumpfüßen Achillotenotomien komplikationslos in Allgemeinanästhesie durchgeführt.

## Befund

Die Indikation zur Sectio caesarea wurde aufgrund des Verdachts eines cephalopelvinen Missverhältnisses bei maternalem Kleinwuchs und einer kindlichen Querlage gestellt. Die Patientin wog bei einer Körpergröße von 125 cm 58 kg und hatte einen Body-Mass-Index von 37,1 kg/m^2^. Im Rahmen der anästhesiologischen Risikoevaluierung zeigte sich kein höhergradiger Verdacht auf das Vorliegen eines schwierigen Atemwegs (Mallampati-Score 2, thyreomentaler Abstand ≥ 6 cm, „upper lip bite test“ 2, Mundöffnung ≥ 4 cm, Kopfreklination problemlos, Kopfrotation eingeschränkt). Die Patientin war mit Unterarmgehstützen eingeschränkt belastbar. Trotz fortgeschrittener Schwangerschaft lagen keine Ödeme der Extremitäten vor. Die kardiopulmonale Belastbarkeit erschien nicht eingeschränkt. Die häusliche Medikation bestand aus Eisen und Magnesium. Im Vergleich zum Rumpf waren die Extremitäten deutlich verkürzt, die Wirbelsäule wies eine ausgeprägte linkskonvexe Skoliose auf. Es bestanden ein Beckenschiefstand und eine Hüftdysplasie.

**Abb. 1:**
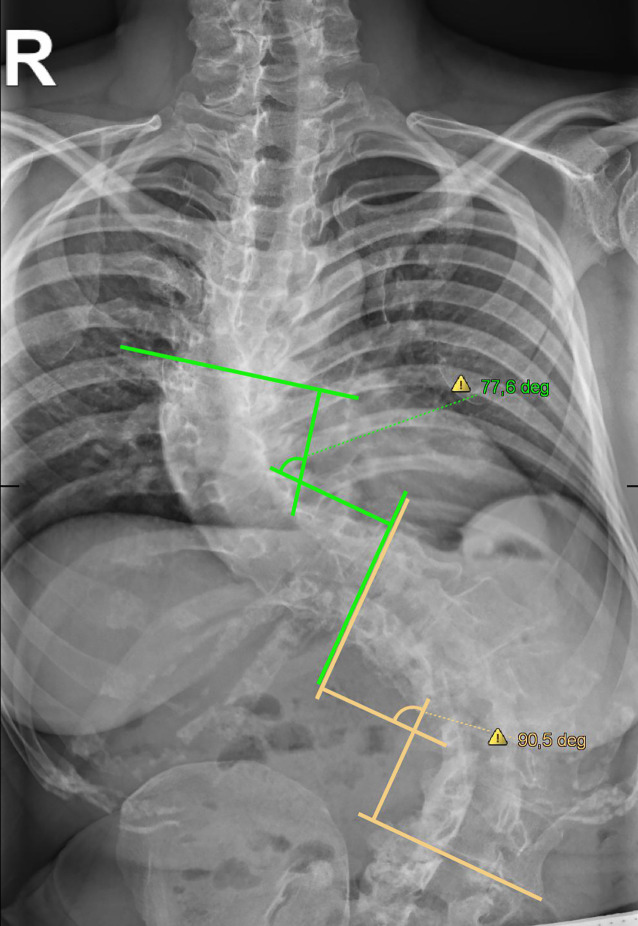
Röntgenbild der Wirbelsäule anterior-posterior mit eingezeichneten Cobb-Winkeln als Maß der Krümmung (thorakal 77,6°, lumbal 90,5°)

In der mitgebrachten Röntgenübersichtsaufnahme der Wirbelsäule (Abb. 1) zeigte sich die linkskonvexe Skoliose mit einer ausgeprägten Torsion. Der Cobb-Winkel, als Maß der sagittalen Wirbelsäulenkrümmung, betrug im Bereich der Brustwirbelsäule 77,6 Grad und im Bereich der Lendenwirbelsäule 90,5 Grad, was insgesamt einer hochgradigen Skoliose entspricht. Alle erhobenen Laborparameter waren im Normbereich.

## Therapie und Verlauf

**Abb. 2:**
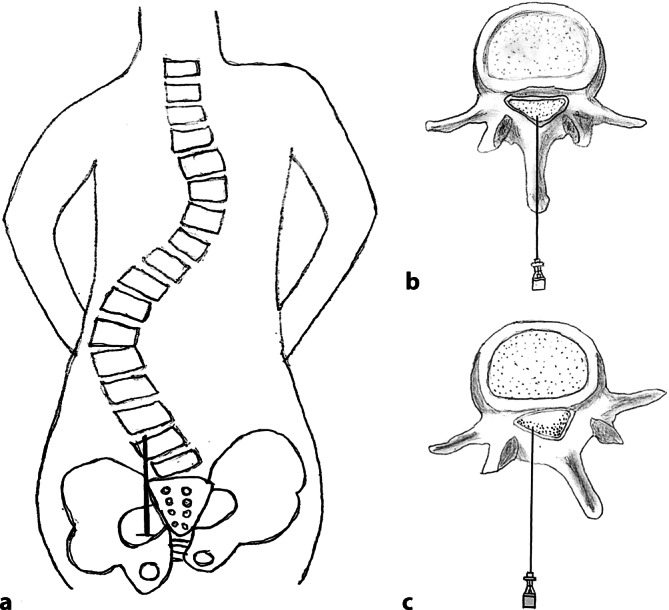
Skizziert ist eine doppelkonvexe Skoliose (**a**) von dorsal. **b** Anatomische Lage eines Lendenwirbelkörpers (Normalbefund). **c** Bei dieser Skoliose sind die Wirbelkörper in der Frontalebene gekippt, in der Transversalebene rotiert und insgesamt deformiert. Auffallend ist die dorsale Anordnung der Cauda equina

Die Sectio caesarea sollte dem Patientenwunsch entsprechend in Spinalanästhesie durchgeführt werden. Nach Lagerung und Desinfektion nach Hausstandard erfolgte zunächst eine Analgesie der Haut sowie der darunterliegenden Strukturen mit 100 mg Mepivacain. Wir verwendeten eine 27-G-Whitacre-Spinalnadel und wählten den Zugang 2 cm paramedian links in gerader Punktionstechnik auf Höhe von L3/4 (Abb. 2). Nach sofort erfolgreicher Spinalpunktion injizierten wir 7 mg Bupivacain, aufgezogen in 7 ml 0,9 %iger NaCl-Lösung plus 1 ml = 5 µg Sufentanil intrathekal. Daraufhin erfolgte die Lagerung auf den Rücken. Die Thermoanästhesie reichte bis Th 6 beidseits. Zur Blutdrucküberwachung nutzten wir neben dem Standardverfahren die kontinuierliche nichtinvasive arterielle Blutdruckmessung mit CNAP® (CNAP®Monitor, CNSystems Medizintechnik GmbH, Graz, Österreich). Zur Vermeidung von Hypotonien wurden insgesamt 5 ml einer Mischung aus Cafedrin/Theodrenalin und 0,9 %iger NaCl-Lösung (2 ml Cafedrin/Theodrenalin auf 8 ml 0,9 %ige NaCl-Lösung) fraktioniert injiziert, sowie 0,5 mg Atropin bei einem Abfall der Herzfrequenz von 110 auf 65 Schläge/min. Nach Freigabe durch die Anästhesie erfolgte eine komplikationslose Schnittentbindung nach Misgav-Ladach [17]. Nach Drehung bei intakter Fruchtblase konnte problemlos ein Junge aus der Beckenendlage entwickelt werden. Der pH-Wert aus der Nabelschnurarterie betrug 7,42 mit einem Base Excess von −1,40 mmol/l. Die Apgar-Werte nach einer, 5 und 10 min betrugen 9/10/10. Das Kind hatte einen Kopfumfang von 40 cm, war 47 cm lang und wog 3305 g. Der postoperative Verlauf war komplikationslos, und die Patientin und ihr Kind konnten am 3. postoperativen Tag nach Hause entlassen werden.

## Diskussion

Die diastrophe Dysplasie ist eine autosomal-rezessiv (Chromosom 5) vererbte Skelettdysplasie mit einer Häufigkeit von 1:100.000, die zu disproportioniertem Kleinwuchs mit hochgradiger Einschränkung der Gelenkbeweglichkeit führt [12]. Ursache sind homozygote Mutationen im Sulfattransporter-Gen (*SLC26A/DTDST*), welche vorwiegend im Knorpel exprimiert werden [2].

Die zur Vorbereitung durchgeführte Recherche bei OrphanAnesthesia über die diastrophe Dysplasie blieb erfolglos. Ebenso gab es keinerlei Daten zur Anwendung einer „High-volume-low-concentration“-SPA bei Kleinwuchs.

Bisher sind lediglich 3 Case Reports in der Literatur veröffentlicht. In 2 Fällen wurde eine Allgemeinanästhesie und in einem Fall eine Periduralanästhesie zur Sectio caesarea durchgeführt. In einem Fall traten Hypoxämien der Mutter in der postoperativen Phase auf [1, 4, 8].

Ein rückenmarknahes Verfahren in Form einer Spinal- oder Periduralanästhesie stellt bei diastropher Dysplasie eine relative Kontraindikation dar. Bei diesem Krankheitsbild kommt es aufgrund der Kyphoskoliose zu Einengungen im Spinalkanal, sodass unklar ist, ob die Dosis des Lokalanästhetikums angepasst werden muss. Außerdem fehlen typische Landmarken zur Durchführung eines rückenmarknahen Anästhesieverfahrens. Die Kyphosen im Bereich der Halswirbelsäule können zu einem erwartet schwierigen Atemweg führen.

Aufgrund der knöchernen Veränderung des Brustkorbs können sowohl die Lungen als auch das Herz in ihrer Funktion eingeschränkt und aufgrund der Disproportionen der Extremitäten eine nichtinvasive Blutdruckmessung unmöglich sein.

Insbesondere aufgrund des Anstiegs der Durchführung neuroaxialer Anästhesieverfahren, aber auch durch Verbesserung der Überwachungsmöglichkeiten, Etablierung von Atemwegsalgorithmen und moderner Atemweg-Devices, konnte die Letalität der Schwangeren bei Sectio caesarea auf 0,15/100.000 in den letzten 40 Jahren in Industrienationen gesenkt werden. Die Spinalanästhesie gilt als Anästhesieverfahren der Wahl. Zu den Vorteilen gegenüber der Allgemeinästhesie zählen das emotionale Erleben des Geburtsereignisses, der fehlende Transfer von Medikamenten über die Plazenta, eine prolongierte Schmerztherapie und die frühzeitige Möglichkeit des Bondings. Gegenüber einer Periduralanästhesie ist der Wirkeintritt deutlich schneller. Des Weiteren zeigt die Periduralanästhesie eine geringere Erfolgsquote als die Spinalanästhesie.

Ein weiterer Vorteil neuroaxialer Verfahren gegenüber einer Allgemeinanästhesie besteht in dem besseren Outcome für Mutter und Kind, gemessen am Apgar-Wert bzw. postpartalen Aufnahmen auf die Intensivstation [5, 9, 13].

Eine Alternative zur klassischen Spinalanästhesie auf Höhe L3/L4 stellt der Taylor-Approach dar. Erstmalig 1940 von A. Taylor beschrieben, handelt es sich hierbei um eine paramediane Punktionstechnik des Subarachnoidalraumes auf Höhe L5/S1. Dieser Zugang wurde ursprünglich in Bauchlage durchgeführt, gilt als lagerungsunabhängig und ist auch bei Patienten mit Skoliose anwendbar. Außerdem beschreiben die Autoren eine höhere Kreislaufstabilität und seltener auftretende postpunktionelle Kopfschmerzen [11].

Die präoperative anästhesiologische Risikoevaluierung bei unserer Patientin zeigte keinen Hinweis auf das Vorliegen eines schwierigen Atemwegs, und anamnestisch verliefen Vornarkosen im Kindesalter in Allgemeinanästhesie problemlos.

Während der Patientenwunsch klar formuliert wurde – Spinalanästhesie –, lagen für alle Anästhesieverfahren erhöhte Risiken vor.

Nach sorgfältiger Risiko-Nutzen-Abwägung und Aufklärung der Patientin entschieden wir uns, eine Spinalanästhesie in High-volume-low-concentration-Technik durchzuführen. Diese ermöglicht einen schnellen Wirkeintritt mit ausreichendem Anästhesieniveau. Jokinen et al. konnte in einer retrospektiven Analyse zeigen, dass die Rate an Verfahrenswechsel mit 0,84 % sehr niedrig ist. Die Hypotonierate wurde mit 48,8 % angegeben, wobei der Vergleich mit der Literatur aufgrund verschiedener Definitionen der Hypotonie erschwert wird [3].

Prospektive Studien, die eine High-volume-low-concentration-SPA mit einer Low-volume-high-concentration-SPA vergleichen, existieren derzeit nicht. Aufgrund eigener Untersuchungen im Rahmen einer abgeschlossenen Promotionsarbeit konnten wir zeigen, dass die Rate an Verfahrenswechseln bei der High-volume-Technik signifikant niedriger als bei der Low-volume-high-concentration-SPA ist. Die Hypotonierate war in beiden Gruppen vergleichbar (59,8 % High volume vs. 64,4 % Low volume) [16].

Die Wirbelsäule ist im Vergleich zu gesunden Patienten v. a. gestaucht und verdreht, aber weniger in ihrer Gesamtlänge reduziert. Aus diesem Grund gingen wir von vergleichbaren Liquorvolumina aus, jedoch von veränderten pathologischen „Innenräumen“ im Spinalkanal. Daher entschieden wir uns, nach interdisziplinärer Konsultation unserer hiesigen Wirbelsäulenchirurgen, höhere Lokalanästhetikavolumina zu verwenden.

Wenk et al. zeigten 2010, wie bei ausgeprägter Skoliose ein thorakaler Periduralkatheter zu platzieren ist, in dem sie den paramedianen Zugang modifizierten [14]. Wir wählten den Zugang auf Höhe L3/4 ca. 2 cm paramedian allerdings in gerader Stichrichtung (Abb. 2).

Wir orientierten uns hierbei an der mitgebrachten Röntgenaufnahme der Wirbelsäule. In der Literatur wurde zur Vorbereitung eines rückenmarknahen Verfahrens eine Magnetresonanztomographie des thorakolumbalen Übergangs durchgeführt [8]. Auffallend ist bei Skoliose die anatomisch veränderte Lage der Cauda equina am dorsalen Rand des Spinalraums (Abb. 2c).

Bei fehlenden anatomischen Landmarken ist eine zuverlässige präoperative Bildgebung der Wirbelsäule sinnvoll. Mittlerweile ebenfalls zum Standard gehörend ist eine sonographisch gesteuerte Punktion. Im Vergleich zur Landmarkentechnik erhöht die ultraschallgesteuerte Punktion signifikant die Trefferquote beim ersten Punktionsversuch. Der Patientenkomfort wird erhöht, Schmerzen während der Punktion sowie postpunktionelle Kopfschmerzen und Rückenschmerzen werden gesenkt [6, 7, 10, 15].

## Fazit

- Trotz ausgeprägter linkskonvexer Skoliose mit Torsion im Rahmen einer diastrophen Dysplasie ist es möglich, durch einen modifizierten paramedianen Zugang eine problemlose Spinalanästhesie durchzuführen.
- Eine Bildgebung der Wirbelsäule sollte vorab vorliegen, und die Punktion sollte ultraschallgesteuert durchgeführt werden.
- Die diastrophe Dysplasie ist ein komplexes Krankheitsbild. Daher ist es notwendig, diese speziellen Patientinnen sorgfältig interdisziplinär vorzubereiten.
